# Implication of 5-HT7 receptor in prefrontal circuit assembly and detrimental emotional effects of SSRIs during development

**DOI:** 10.1038/s41386-020-0775-z

**Published:** 2020-07-20

**Authors:** Jimmy Olusakin, Imane Moutkine, Sylvie Dumas, Evgeni Ponimaskin, Eleni Paizanis, Mariano Soiza-Reilly, Patricia Gaspar

**Affiliations:** 1 Institut du Fer à Moulin, INSERM, UMR-S, 1270 Paris, France; 2 Sorbonne Université, Paris, France; 3 Oramacell, Paris, 75006 France; 4 Hannover Medical School (MHH), Hannover, Germany; 5 INSERM, U1075 COMETE UNICAEN, University of Caen Normandie, Caen, France; 6 Instituto de Fisiología, Biología Molecular y Neurociencias (IFIBYNE), CONICET, Universidad de Buenos Aires, Buenos Aires, Argentina; 7 Institut du Cerveau et de la Moëlle, CNRS UMR 7225-Inserm U1127, Paris, France; 8 Present Address: Département des Neurosciences Fondamentales, College of Médecine, University of Geneva, 1 rue Michel Servet, 1211 Geneva, Switzerland

**Keywords:** Synaptic development, Depression

## Abstract

Altered development of prefrontal cortex (PFC) circuits can have long-term consequences on adult emotional behavior. Changes in serotonin homeostasis during critical periods produced by genetic or pharmacological inactivation of the serotonin transporter (SERT, or Slc6a4), have been involved in such developmental effects. In mice, selective serotonin reuptake inhibitors (SSRIs), administered during postnatal development cause exuberant synaptic connectivity of the PFC to brainstem dorsal raphe nucleus (DRN) circuits, and increase adult risk for developing anxiety and depressive symptoms. SERT is transiently expressed in the glutamate neurons of the mouse PFC, that project to the DRN. Here, we find that 5-HTR7 is transiently co-expressed with SERT by PFC neurons, and it plays a key role in the maturation of PFC-to-DRN synaptic circuits during early postnatal life. 5-HTR7-KO mice show reduced PFC-to-DRN synaptic density (as measured by array-tomography and VGLUT1/synapsin immunocytochemistry). Conversely, 5-HTR7 over-expression in the developing PFC increased PFC-to-DRN synaptic density. Long-term consequences on depressive-like and anxiogenic behaviors were observed in adults. 5-HTR7 over-expression in the developing PFC, results in depressive-like symptoms in adulthood. Importantly, the long-term depressive-like and anxiogenic effects of SSRIs (postnatal administration of fluoxetine from P2 to P14) were not observed in 5-HTR7-KO mice, and were prevented by co-administration of the selective inhibitor of 5-HTR7, SB269970. This study identifies a new role 5-HTR7 in the postnatal maturation of prefrontal descending circuits. Furthermore, it shows that 5-HTR7 in the PFC is crucially required for the detrimental emotional effects caused by SSRI exposure during early postnatal life.

## Introduction

Enhanced vulnerability to emotional disorders such as anxiety and depression can result from altered developmental trajectories during early life [1]. This, in turn, can be related to a combination of genetic and environmental factors including early-life stress, malnutrition, or drug exposure. In these conditions, perturbed serotonin (5-HT) signaling has been implicated as a causal agent because of its impact on brain development [2–4].

One of the most remarkable and clinically relevant examples of 5-HT’s developmental effects is the exposure of human fetuses or infants to selective serotonin reuptake inhibitors (SSRIs) [5]. Contrasting with their antidepressant effects in adulthood, fluoxetine, as other SSRIs, when administered during early life, have long-term effects on adult emotional behavior including enhanced anxiety and depressive-like phenotypes [6]. Similarly, serotonin transporter (SERT) knock-out mice show increased anxiety and depressive-like behaviors [7, 8], and humans carrying the hypofunctional s-allele of the SERT gene have increased risk to mood disorders after early adversity [9, 10]. Overall, these observations indicated that increased 5-HT signaling obliterates normal physiological balances during a critical period when the neural circuits that underlie stress-related behaviors are being remodeled by activity-dependent processes [11].

The difference in the action of SSRIs in development and adults could be explained by changes in cellular targets of SSRIs at different critical periods of life. While SERT is expressed exclusively in the 5-HT-producing neurons of the raphe nuclei in adult brain, it has a broader expression during fetal and early postnatal life in rodents [12, 13] and primates including humans [[14, 15]; http://www.brainspan.org]. This suggested that SERT has specific developmental functions, in particular to buffer 5-HT accumulation and thereby controlling 5-HT receptor (5-HTR) activation [3, 16]. Recently, a key neuronal circuit involved in mood control, the descending prefrontal projection to raphe neurons [17–19] has been found to transiently express SERT, during the first 2 weeks of life. SERT is expressed in layer 5–6 glutamate projection neurons of the prefrontal cortex (PFC), in particular those projecting to the dorsal raphe nucleus (DRN). Genetic ablation or pharmacological blockade of SERT function in these neurons alters the glutamatergic synapse development [20]. We reasoned that 5-HTRs expressed simultaneously with SERT in the PFC, would be likely candidates to mediate these developmental effects. Based on gene expression profiling we identified the 5-HTR7 as a possible candidate. The 5-HTR7 promotes axon growth and synaptogenesis in vitro [21–23], suggesting a role in neural circuit development and structural plasticity [24]. Moreover, 5-HTR7 has been implicated in mood control, based on pharmacological studies showing that antagonizing 5-HTR7 attenuates anxiety and depression-like phenotypes [25–28]. Furthermore, a variant of 5-HTR7 has been associated to a better response to SSRIs in bipolar patients [29].

In the present study, we used a combination of genetic, pharmacological and high-resolution imaging approaches to evaluate the developmental role of 5-HTR7 in the synaptic wiring of the PFC-to-DRN circuit and adult emotional behavior. We provide converging evidence that 5-HTR7 is crucially required for the detrimental developmental effects of SSRIs on the PFC circuit assembly, with a direct impact on adult emotional control.

## Methods and materials

### Animals

Animal care and experiments were conducted in accordance with the institutional guidelines. All procedures have been approved by the ethical committee of the region Ile de France (Comité Darwin, agreement 09047.04). C57BL/6 (Janvier, France) and 5-HTR7 knockout (5-HTR7^−/−^) mice [26, 30], maintained on a C57BL/6 background, were used. At arrival, pregnant females were group-housed by 2 and litter sizes were homogenized from 8–14 animals with both sexes represented. After weaning at P21, all mice were group-housed by sex (4–5 per cage), and kept under standard laboratory conditions (22 ± 1 °C, 60% relative humidity, 12–12 h light–dark cycle, food and water *ad libitum*) in ventilated racks. Male and female mice were used, and the number of individuals used are provided in Figure legends.

### Drug administration

Fluoxetine hydrochloride (FLX, Tocris, UK) and SB269970 (Abcam, #ab120508) were used. FLX was diluted in 3% sucrose when administered orally, or in saline (0.9% NaCl) when administered subcutaneously. Subcutaneous injections (1 ml/kg) were carried out only in experiments where pups received a second treatment that cannot be orally administered (i.e., SB269970) [31]. SB269970 was administered at 10 mg/kg twice daily based on previous studies [28].

Mixed litters born from 5-HTR7^−/−^ x 5-HTR7^+/−^ breedings were given FLX (10 mg/kg/day) or sucrose (3% in water) orally from P2 to P14. Litters were separated from their dam, weighed, and fed either the FLX/sucrose or sucrose solution with a P10 pipette, before being returned to their dams. As pups very readily absorb the sucrose, the entire procedure lasted <5 min per litter, and had no visible consequences on maternal care to pups. Genotyping was performed at weaning using primers to detect the neo cassette and the deleted exon 2 gene (Supplementary Table S1).

Litters of C57BL/6 mice were randomly assigned to: Saline (Control; 0.9% NaCl/24 h); Fluoxetine (FLX; 10 mg/kg/24hs); FLX (10 mg/kg/24 h) plus SB269970 (10 mg/kg/12 h) (in this condition, FLX was co-administered with one of the SB269970 injections); and SB269970 (10 mg/kg/12 h). Drugs and vehicle were administered s.c. from P2 to P14.

### RT-qPCRs

Brains of mice euthanized at P1, P7, P14, and P60 (*n* = 5/age) were extracted and kept on ice while PFCs were dissected out. 2–3 brains were pooled for each age analyzed. Samples were directly processed for RNA isolation with Trizol reagent (ThermoFisher, France, #15596026) following standard protocols. Genomic DNA was cleared using DNaseI (ThermoScientific, France, #EN0521) and RT-PCR was done with SuperScript II kit (Invitrogen, USA, #18064014) using ThermoScientific SYBR Green Mix. Primers are provided in Supplementary Table S1.

### In situ hybridization (ISH)

The following probes were used for 5-HTR7 mRNA: NM_008315.3 sequence 602–1372, Slc6a4 mRNA: NM_013034.4 sequence 1192–2047, and GFP: NM_013645 sequence 74–588.

Brains of P0, P7, P14, P21, and adult mice (*n* = 3–4/age; P0 = birth) were rapidly frozen in isopentane at −30 °C and stored at −80 °C. Tissue blocks were sectioned at 15 μm on a cryostat, and dried at −20 °C for at least 60 min.

Colorimetric ISH was carried out as described previously [32], using a 5-HTR7 DIG-labeled probe, phosphatase-coupled anti-DIG antibodies (1:1000), and developed with NBT/BCIP. For double labeling experiments the RNAscope Multiplex Fluorescent Reagent Kit (Advanced Cell Diagnostics, France) was used. Probes were designed by Advanced Cell Diagnostics to target: *Slc6a4* (#315851) and *5-HTR7* (#401321-C3). ISH was performed according to the protocol of the RNAscope Multiplex Fluorescent Reagent Kit v1 (#320851). Before mounting, sections were incubated with DAPI. ISH imaging was done in a Zeiss Axioscan microscope at 20X, while FISH images were acquired with a confocal microscope at 20X and 63X.

### 5-HTR7-EGFP construct

The construction of the 5-HTR7 fused to EGFP has been described previously [33]. The 5-HTR7-EGFP fusion construct was then cloned into an AAV plasmid under control of the neuron specific synapsin promoter [34]. This plasmid was used for production of the rAAV8 (Vector Core, Univ. North Carolina, USA) coding for *5-HTR7-EGFP*. AAV-9-hSyn-eGFP-WPRE-bGH (Penn Vector Core, USA) was used as sham. The AAV8-9 serotypes were chosen based on the rapidity and efficiency of neuronal transfection [35].

### PFC viral injections

Newborn mice were separated from their dams and randomly assigned to 5-HTR7 overexpression group (rAAV8-Syn(H1-2)-5-HTR7-EGFP) or sham group (AAV-9-hSyn-EGFP-WPRE-bGH). Titer ranges were of 10^12^–10^13^ particles/ml. P1 pups were cold-anesthetized and bilaterally injected (50 nl each) using a pulled glass capillary mounted on a hydraulic micromanipulator as before [20]. The whole procedure takes about 5–10 min per pup. After surgeries, pups were warmed and rapidly returned to their home cages with the mothers.

### Array tomography

Fixed brain tissue containing the DRN were processed for array tomography as previously [36, 37]. Tissue was ultrasectioned in series of 25 100 nm-thick sections in an ultramicrotome (Leica) [36, 37]. Polyclonal antibodies anti-Tryptophan hydroxylase (sheep, 1:200; Millipore, USA; AB1541), anti-VGLUT1 (guinea pig, 1:1000; Millipore, USA; AB5905) and anti-Synapsin 1a (rabbit, 1:200; Cell Signaling Technology, MA, USA; D12G5 XP, #5297S) were used [37]. Fluorescent-conjugated secondary antisera raised in donkey (Alexa 488,647 and CY3, 1:100; Jackson ImmunoResearch, USA) were applied for revelation. Sections were imaged in a Leica DM6000 fluorescence microscope using a 63X NA 1.4 oil objective. Serial images were processed and analyzed using Fiji as described previously [36, 37].

### Behavioral studies

Behavioral measurements started at P80 in the following sequence: (1) Open field (OF), (2) Splash test (ST), (3) Novelty-suppressed feeding (NSF), (4) Forced-swim test (FST) and 5) Locomotion. 2-day interval was set between the OF and ST and 7-day interval between the NSF, FST and locomotor tests to minimize interferences. Behaviors were done during the light cycle (10 a.m.–5 p.m.) as previously [20, 35].

### Open field

Mice were allowed to explore brightly lit (400–500 lux) chambers [50 cm × 50 cm × 45 cm] equipped with an infrared camera (B/W-CCD with CCTV lens 2.8–12 mm) connected to a computer. Mice were placed in the center of the chamber and allowed to explore for 9 min. Ambulatory distances, time spent and distance traveled in the center of the arena were measured with Viewpoint software.

### Splash test

ST was carried out in a fresh cage as before [35], where mice were individually sprayed on their back twice with 20% sucrose solution in water and placed in a corner of their home cage. The latency to start grooming was  manually recorded.

### Novelty-suppressed feeding

NSF was done in a plastic box [50 × 80 × 20 cm] with the floor covered with 3 cm of wooden bedding. 24 h before testing, mice were food deprived in their home cage. Mice were individually weighed before and after food deprivation to determine % weight loss. During the test 2 food pellets were placed on a circular white filter paper (12 cm diameter) located in the center of the arena. Mice were placed in a corner of the box and latencies to approach pellets and feed were recorded [20]. After the test, the weight of pellet consumption in the home cage during 5 min was registered.

### Forced-swim test

FST was carried out in a glass cylinder (40 cm × 20 cm diameter) filled half-way with water (23–24 °C). Mice were tested over 2 days for 6 min each day after which they were dried and returned to their home cages. All swim sessions were videotaped and time of immobility during the second day session was quantified [20].

### Locomotor activity

Mice were introduced into a circular corridor (4.5 cm width, 17 cm diameter) equipped with four infrared beams equidistantly located every 90° (Imetronic, Pessac, France). Locomotor activity was recorded for 30 min [20].

### Statistical analyses

Sample size was established by previous pilot and published studies. Mice of each genotype/sex were randomly assigned to different studies. Data distribution was controlled for normality by Q-Q plots, and when ANOVA was applied the homogeneity of variances of the data was tested. For array tomography, the results obtained from two stacks were averaged to generate a mean value per mouse. Data were analyzed using multifactorial ANOVA or two-tailed Student’s t-test, using Prism 6 and IBM SPSS 20. Data are expressed as mean ± SEM. Significance was established at *p* < 0.05.

## Results

### 5-HTR7 is expressed in PFC-SERT+ neurons during early postnatal development

**Fig. 1:**
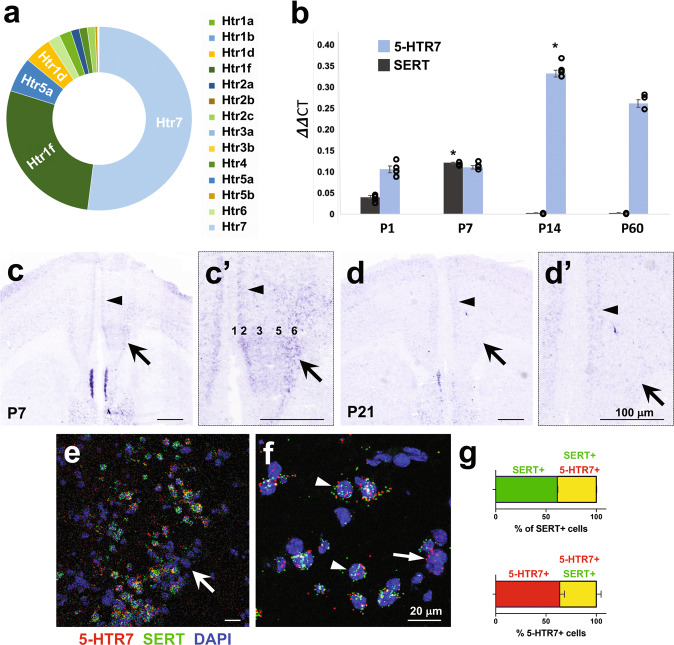
5-HTR7 expression in the mouse mPFC is developmentally regulated during the early postnatal period. **a** Relative proportion of 5-HT receptor genes (*Htr*) expressed in PFC-SERT + neurons at P7, as revealed by RNA sequencing profiling of FACS-isolated SERT^Cre^::EGFP neurons from the mPFC. **b** Time course of 5-HTR7 and SERT mRNA expression by q-PCR on mPFC tissue dissected at P1, P7, P14 and P60 (*n* = 3–4 per age). *p < 10^−6^ for SERT at P7 vs. all ages (*F*_3,7_ = 314.103; *p* < 10^−7^), and 5-HTR7 at P14 vs. P1–P7 (*F*_3,7_ = 129.497; *p* < 10^−5^) after ANOVA followed by Tukey’s test. **c**, **d** In situ hybridization of 5-HTR7 in the mPFC at P7 (c,c’) and P21 (d,d’) illustrating a laminar shift in the distribution of the receptor during the early postnatal period. Abundant expression of 5-HTR7 is present in layers 5–6 at early ages (arrows), however by P21 this expression remains only in superficial 2–3 layers (arrowheads). **e**, **f** RNAscope double fluorescent in situ hybridization in the mPFC at P7 (**e**, arrow), showing nuclear (DAPI, blue) co-localization of 5-HTR7 mRNA (red) with SERT mRNA (green) expression (arrowheads). Some of these mPFC neurons present 5-HTR7 mRNA but not SERT expression (**f**, arrow). **g** Quantitative analysis of the percentage of mPFC neurons co-expressing SERT (green) and 5-HTR7 (red) mRNAs.

The neurons of the PFC that transiently express SERT during early postnatal mouse development constitute a very specific subpopulation of glutamate projection neurons of layers 5 and 6. To determine which 5-HTRs are present in PFC-SERT+ neurons, we analyzed data obtained in a previous RNA sequencing study of FACS-isolated PFC-SERT+ neurons at P7 [20]. This analysis indicated that the 5-HTR7 has the highest expression in PFC-SERT+ neurons, followed by the 5-HTR1F, 5-HTR5A, and 5-HTR1D, whereas other classical cortical receptor subtypes like the 5-HTR1A or 5-HTR2A have only a minor expression (Fig. 1a). Real-time quantitative PCR (qPCR) studies corroborated the coincidence of 5-HTR7 and SERT expression in the PFC at P7 (Fig. 1b). Furthermore, extended qPCR analysis at different postnatal ages showed that 5-HTR7 mRNA is already present in the PFC by P1, that this expression increases by P7 and peaks at P14, with a moderate reduction by P60 (Fig. 1b). In situ hybridization (ISH) results revealed layer-specific expression profiles in the PFC, indicating that 5-HTR7 is abundantly expressed in cortical neurons of layer 2 and layers 5–6 at P0, P7 and P14 (Fig. 1c,c’; Supplementary Fig. S1). However, at later ages (P21), the 5-HTR7 mRNA expression decreased substantially in deep layers, while expression in layer 2 remained moderate (in the mPFC) (Fig. 1d,d’; Supplementary Fig. S1) to high (in the anterior cingulate cortex) (Supplementary Fig. S1). To further explore the 5-HTR7 and SERT co-expression in PFC-SERT+ neurons, we performed double labeling fluorescent ISH. This study showed robust 5-HTR7 and SERT co-expression in cortical neurons of layers 5 and 6 at P7 (Fig. 1e, f). We found that 40% of SERT+ neurons in the deep layers of the mPFC contain 5-HTR7 transcripts, indicating the presence of a subpopulation of SERT+ neurons expressing the receptor by P7. In addition, a similar proportion of 5-HTR7-expressing neurons within the same cortical region contained SERT transcripts (Fig. 1g). Overall, these observations indicated that 5-HTR7 is expressed in the mPFC, with a strong transient expression in deep cortical layers during the first weeks of postnatal life, and is co-expressed with SERT in these neurons.

### 5-HTR7 regulates the PFC-to-DRN circuit assembly

**Fig. 2:**
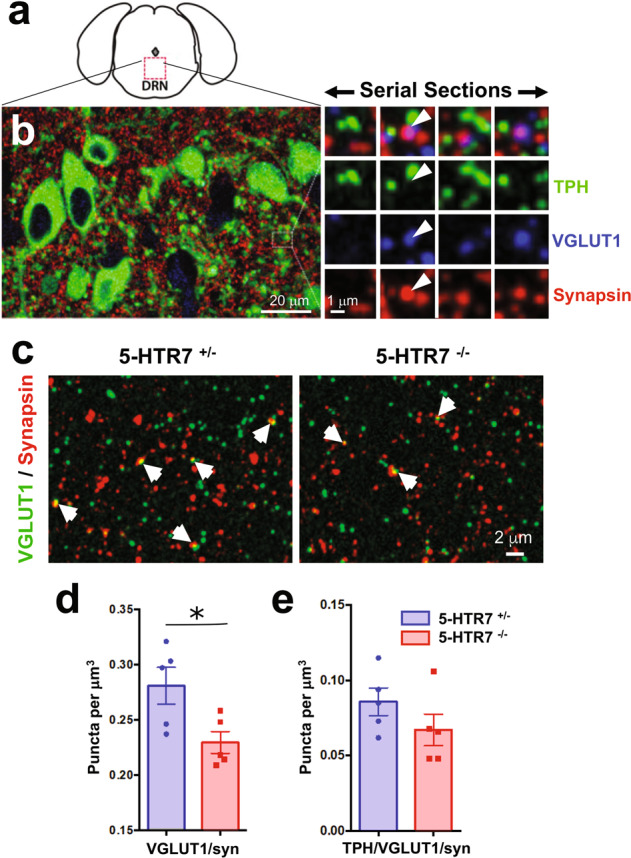
Genetic ablation of the 5-HTR7 diminishes the synaptic afferents of the PFC-to-DRN circuit. **a** Diagram indicates the region sampled for the array tomography quantitative analysis. **b** Array tomographic render from seven 100 nm thick sections immunolabeled for tryptophan hydroxylase (TPH, green), synapsin (red) and the vesicular glutamate transporter type (VGLUT1, blue). The zoomed-in area in the right panel shows four serial sections in which VGLUT1 and synapsin co-label the same axon bouton (arrowhead), in close apposition to TPH + neuronal profiles. **c** Representative high-resolution array tomography image of double labeled puncta against VGLUT1 and synapsin (arrows) in a 100 nm thick section of the DRN from 5-HTR7^+/−^ and 5-HTR7^−/−^ mice. Quantitative analysis of PFC glutamatergic synaptic afferents in the DRN of 5-HTR7^+/−^ and 5-HTR7^−/−^ mice. All VGLUT1/synapsin+ puncta (*t*_8_ = 2.680; *p* < 0.03) (**d**), and VGLUT1/synapsin+ puncta in contact with TPH + neurons (*t*_8_ = 1.348; *p* = 0.22) (**e**) are shown.

Fluoxetine (FLX) exposure during a critical period of postnatal age (P2–P14) produces an increased number of PFC glutamatergic synapses onto 5-HT and GABA neurons in the dorsal raphe nucleus (DRN) [20]. To determine whether 5-HTR7 is involved in this effect, we examined the consequences of 5-HTR7 loss and gain of function. The synaptic density of PFC glutamatergic inputs to the DRN was measured by the high-resolution immunofluorescence technique array tomography [38]. This microscopy technique allows identifying cortical axon terminals containing the vesicular glutamate transporter type 1 (VGLUT1) [37, 38]. Cortical afferents to the DRN arise mainly from the PFC [39–41]. Because of this, VGLUT1 immunolabelling can be used as a readout of PFC synaptic afferents, together with the co-labeling of synapsin 1a, a general marker for synaptic boutons [37, 42]. In addition, tryptophan hydroxylase (TPH) immunolabeling determines the presence of morphological contacts of synaptic boutons to 5-HT neurons [37, 42] (Fig. 2a, b). Array tomography quantitative analyses of VGLUT1/synapsin double labeled puncta in 5-HTR7^−/−^ mice (38,900 puncta analyzed) showed a significant reduction of 18% (*t*_8_ = 2.680; *p* < 0.03) compared with littermate controls (5-HTR7^−/+^ mice; 29,938 puncta analyzed) (Fig. 2c, d), suggesting a role in PFC-to-DRN circuit formation of 5-HTR7. The analysis of VGLUT1/synapsin double labeled boutons onto 5-HT neurons showed a non-significant trend to decrease, suggesting that total changes observed (Fig. 2d) would have a preferential impact on non-5-HT neurons of the DRN (Fig. 2e; a total of 11,025 and 8,553 puncta analyzed for 5-HTR7^−/+^ and 5-HTR7^−/−^ mice, respectively).

**Fig. 3:**
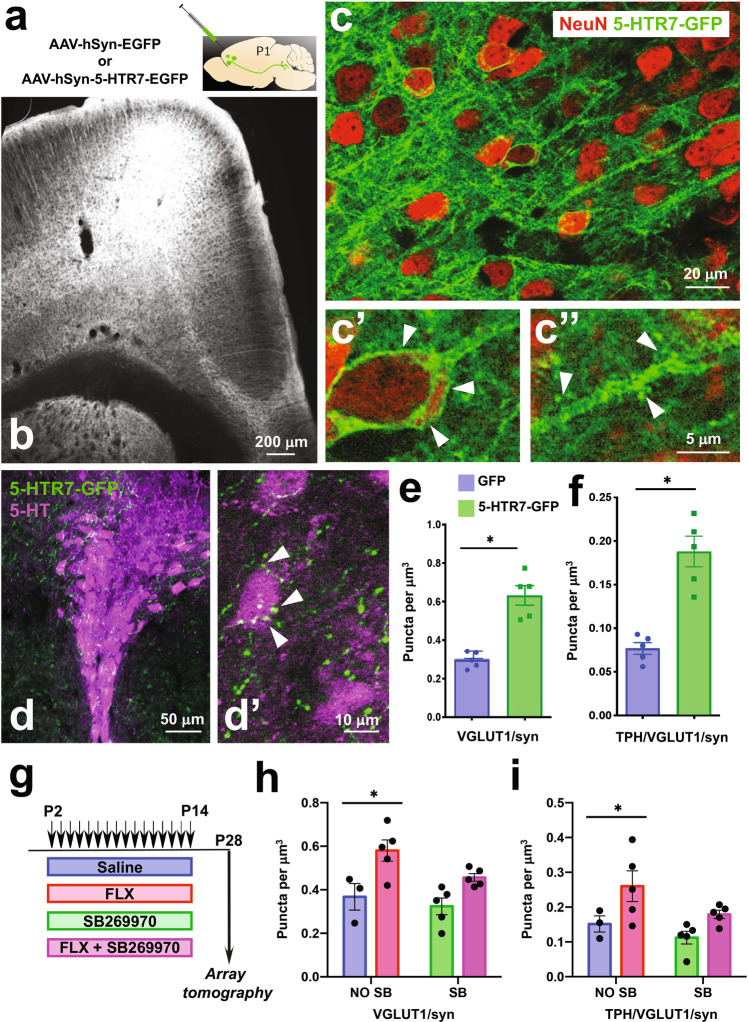
Developmental role of 5-HTR7 in the synaptic wiring of the PFC-to-DRN circuit. **a**, **b** Bilateral injections of the AAV-hSyn-5-HTR7-EGFP or AAV-hSyn-EGFP were carried out in the PFC of C57BL/6 mice at P1. Coronal section through the PFC at P12 shows heavy 5-HTR7-GFP expression in deep cortical layers (**b**). **c** High power confocal images of pyramidal neurons expressing the construct encoding 5-HTR7-GFP (green) with NeuN counterstaining (red). The 5-HTR7-GFP localizes at the cell membrane of pyramidal cell somas (**c′**) and dendritic processes including spines (**c′′**). 5-HTR7-GFP labeling is visible in axon terminals in the DRN (green) (**d**), often associated with TPH + neurons (5-HT, magenta) (**d′**). Quantification of VGLUT1/synapsin+ axonal boutons in the DRN of P28 mice that received injections of either sham (GFP; *n* = 5) or 5-HTR7-EGFP (*n* = 5) viral constructs in the PFC at P1. All VGLUT1/synapsin+ puncta (**e**), and VGLUT1/synapsin+ puncta in contact with TPH+ neurons (**f**) are shown. *p < 0.001. **g**–**i** Four groups of C57BL/6 mice were administered subcutaneously with saline (*n* = 3), FLX (10 mg/kg/day; *n* = 5), FLX (10 mg/kg/day) + SB269970 (10 mg/kg/12 h) (*n* = 5) or SB269970 (10 mg/kg/12 h; *n* = 5) during the critical period (P2–P14) . Array tomography quantitative analyses were carried out in the DRN at P28 in all four experimental groups (**g**). Density of VGLUT1/synapsin+ puncta (**h**) [total number of puncta analyzed for saline (21,510), FLX (45,166), FLX+ SB269970 (35,215) and SB269970 alone (26,892)], and VGLUT1/synapsin+ puncta related to TPH + neurons (**i**) [total number of puncta analyzed for saline (7059), FLX (20,011), FLX+ SB269970 (14,563) and SB269970 alone (8907)]. *p < 0.001 and *p* < 0.01 for **h** and **i**, respectively, FLX vs. all treatments.

To further interrogate the role of 5-HTR7 in the development of PFC neurons we selectively over-expressed the full length 5-HTR7 with the C-terminal fused to GFP under the control of the synapsin promoter [33]. Previous studies from our group showed that this promoter preferentially drives AAV transduction in pyramidal glutamate projection neurons of the mPFC [35]. Bilateral injections of AAV8-hSyn-5-HTR7-EGFP in the PFC were done at P1 (Fig. 3a). Histological control showed expression exclusively in the frontal pole including orbital, prelimbic and infralimbic regions of the PFC, with a large number of transduced neurons in layer 5–6 neurons at P7 (Fig. 3b). The GFP-labeling was concentrated in the neuronal membranes, within the somatodendritic compartment (Fig. 3c,c′), and in dendritic spines (Fig. 3c′′) as well as well as in PFC efferent axons. Importantly, in the brainstem, labeling was noted in axon terminals within the DRN, in close proximity to 5-HT labeled neurons (Fig. 3d,d′). Next, we corroborated the virally-induced over-expression levels of the 5-HTR7 mRNA in the PFC by qPCR at P7. The viral construct over-expressed the 5-HTR7 mRNA about 25 times above the expression levels of sham controls (injected with AAV-9-hSyn-EGFP-WPRE-bGH). Further, such increase in 5-HTR7 expression levels was not longer evidenced in adulthood (Supplementary Fig. S2). Using these tools, we observed that 5-HTR7 over-expression in the PFC caused a significant increase in the density of VGLUT1/synapsin double labeled puncta in the DRN when compared with sham control mice (t_8_ = 6.185; *p* < 0.0003) (a total of 54,922 and 34,267 puncta analyzed for 5-HTR7-over-expressing and sham mice, respectively) (Fig. 3e). This effect was also evident when analyzing the double labeled synaptic puncta in morphological contact with TPH-positive neurons (*t*_8_ = 5.895; *p* < 0.0004) (17,763 and 8735 puncta analyzed for 5-HTR7-overexpressing and sham mice, respectively) (Fig. 3f).

Next, we asked whether antagonizing the 5-HTR7 may prevent the morphological synaptogenic effects of FLX exposure during the postnatal critical period. Wild-type mouse pups were administered with FLX or saline, alone or together with the selective 5-HTR7 antagonist SB269970 [31] daily from P2 to P14 (Fig. 3g). Two-way ANOVA results showed no significant interaction between FLX/SAL treatment and SB269970 treatment (*F*_1,14_ = 0.890; *p* = 0.36). Confirming our previous observations, the density of VGLUT1/synapsin double labeled terminals was increased in the FLX-treated mice when compared with the saline-treated controls (Main Effects: *F*_1,14_ = 16.773; *p* < 0.001) (Fig. 3h). This difference was abolished when SB269970 was co-administered with FLX or SAL (Main Effects: *F*_1,14_ = 3.934; *p* = 0.067) (Fig. 3h). Similarly, associations of VGLUT1+ synaptic boutons with TPH-positive neurons were also increased by FLX treatment (Main Effects: *F*_1,14_ = 8.877; *p* < 0.01), but these differences disappeared when administering SB269970 (Main Effects: *F*_1,14_ = 4.198; *p* < 0.06) (Fig. 3i).

Overall, these experiments indicate that 5-HTR7 controls the formation of efferent PFC projections to the brainstem DRN during early postnatal life, highlighting the requirement of 5-HTR7 for the morphogenetic effects of postnatal FLX exposure on the PFC-to-DRN synaptic circuit.

### 5-HTR7 engagement in shaping adult emotional behaviors

**Fig. 4:**
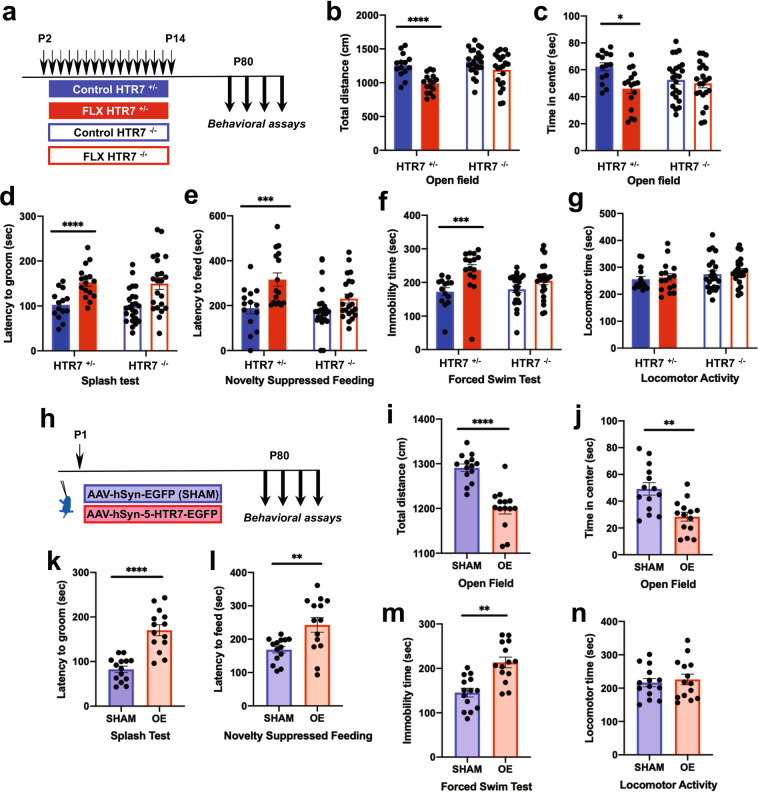
Engagement of 5-HTR7 in shaping adult emotional behaviors. **a** 5-HTR7^+/−^ and 5-HTR7^−/−^ mice were treated orally with FLX (10 mg/kg/day in 3% sucrose solution; *n* = 16 and *n* = 22 for 5-HTR7^+/−^ and 5-HTR7^−/−^ mice, respectively) or vehicle (3% sucrose solution; *n* = 14 and *n* = 24 for 5-HTR7^+/−^ and 5-HTR7^−/−^ mice, respectively) during the critical period (P2–P14), to evaluate emotional behaviors in the adulthood (from P80). **b** Total distance traveled in the Open Field test (OF) (Main Effects treatment: *F*_1,72_ = 17.606; *p* < 0.0001), and time spent in the center of the arena (**c**) (Intera**c**tion treatment x genotype: F_1,72_ = 3.978; *p* < 0.05 in 5-HTR7^+/−^ mice, FLX vs. control: *F*_1,28_ = 10.954; *p* < 0.003). **d** Latency to groom in the Splash Test (ST) (Main Effects treatment: *F*_1,72_ = 22.385; *p* < 0.0001). **e** The latency to feed in the Novelty Suppressed Feeding Test (NSF) (Main Effects treatment: *F*_1,72_ = 13.619; *p* < 0.0004). **f** Immobility time in the Forced Swim Test (FST) (Main Effects treatment: *F*_1,72_ = 13.209; *p* < 0.001). **g** Locomotor activity in a circular path (Main Effects treatment: *F*_1,72_ = 0.451; *p* = 0.50). **h** C57BL/6 mice were bilaterally injected with AAV-hSyn-5-HTR7-EGFP (OE; n = 14) or AAV-hSyn-EGFP (Sham; *n* = 14) in the PFC at P1. Behavioral measurements were carried out in these mice in the OF (**i, j**), ST (**k**), NSF (**l**) and FST (**m**), and locomotor activity (**n**), starting at P80. **p < 0.005 and ****p < 0.0001 in **i**–**n**.

To assess the developmental role of 5-HTR7 in adult emotional phenotypes, we generated new cohorts of the different experimental groups described above to examine behavioral effects in adulthood, focusing on anxiogenic and depressive-like behaviors. Heterozygous breeding was used to generate cohorts of 5-HTR7 ^+/+^; 5-HTR7^+/−^ and 5-HTR7^−/−^ mice. Adult behavioral testing of these mice did not reveal genotype effects in any of the tests used: locomotor activity, exploration of an open arena (OF), latency to groom in the splash test (ST), latency to feed in the novelty suppressed feeding (NSF), and immobility time in the forced swim test (FST) (Supplementary Fig. S3). These observations confirmed the lack of an anxiety phenotype of the 5-HTR7^−/−^ mice, but did not reproduce the reduced immobility time in the FST observed in previous studies [26, 43]. Next, we asked whether the 5-HTR7 could have a role in the adult emotional alterations induced by postnatal exposure to FLX. To this end, 5-HTR7^−/−^ and 5-HTR7^+/−^ mice were administered with FLX or vehicle from P2 to P14 (Fig. 4a). Two-way ANOVA results showed no interaction between genotype and treatment factors in the total distance traveled in the OF (*F*_1,72_ = 2.656; *p* = 0.108) (Fig. 4b). In contrast, FLX-treated mice showed decreased exploration of the arena (Main Effects: *F*_1,72_ = 17.606; *p* < 0.0001), with a robust genotype effect (Main Effects: *F*_1,72_ = 7.406; *p* < 0.008) (Fig. 4b). The results of time spent in the center of the OF arena showed a significant genotype x treatment interaction (*F*_1,72_ = 3.978; *p* < 0.05). Simple effects indicated a reduction in the time exploring the center of the arena only in FLX-treated 5-HTR7^+/−^ mice in comparison to control treated littermates (*F*_1,28_ = 10.954; *p* < 0.003). (Fig. 4c). No differences between treatments or genotypes were observed in total distance traveled in the center (Supplementary Fig. S4a). Analysis of latency to groom in the ST showed a non-significant factor interaction (*F*_1,72_ = 0.002; *p* = 0.96). However, FLX treatment increased the latency to groom (Main Effects: *F*_1,72_ = 22.385; *p* < 0.0001) (Fig. 4d), and these differences appeared more pronouncedly in 5-HTR7^+/−^ mice, however this effect did not reach significance (Main Effects: *F*_1,72_ = 0.053; *p* = 0.82). Similarly, in the NSF, we did not find a significant factor interaction (*F*_1,72_ = 2.777; *p* = 0.10). In contrast, FLX-treated mice showed an increased latency to feed (Main Effects: *F*_1,72_ = 13.619; *p* < 0.0004) (Fig. 4e), and this effect was more robust in 5-HTR7^+/−^ mice, although not significant (Main Effects: *F*_1,72_ = 3.596; *p* = 0.06). On the other hand, we did not find changes in the percentage of weight loss or food consumption in the home cage among experimental groups (Supplementary Fig. S4b,c). Evaluation of the floating time in the FST also showed a non-significant interaction (*F*_1,72_ = 2.540; *p* = 0.12). In this case, FLX-treated mice showed an increase in the floating time (Main Effects: *F*_1,72_ = 13.209; *p* < 0.001) that was more evident in 5-HTR7^+/−^ mice (Fig. 4f), although did not reach statistical significance (Main Effects: *F*_1,72_ = 1.035; *p* = 0.31). Locomotor activity in a circular path was not affected either by the treatment or genotype (Interaction: *F*_1,72_ = 0.023; *p* = 0.88; Main Effects treatment: *F*_1,72_ = 0.451; *p* = 0.50; Main Effects genotype: *F*_1,72_ = 2.414; *p* = 0.13) (Fig. 4g).

Because experiments in 5-HTR7^−/−^ mice do not allow assessing the tissue-specific spatiotemporal requirement of 5-HTR7 expression, we used the viral over-expression approach described previously to specifically interrogate the developmental role of PFC 5-HTR7 in shaping adult emotional behaviors. We compared adult mice that had been injected at P1 with the viral construct encoding for 5-HTR7-GFP, with those injected with the viral construct encoding only for the GFP reporter (Fig. 4h). Behavioral analysis of these mice revealed a reduced exploration and time spent in the center of the arena in the OF (*t*_26_ = 6.222; *p* < 0.0001 and *t*_26_ = 3.638; *p* < 0.002, respectively) (Fig. 4i, j), without changes in distances traveled in the center (Supplementary Fig. S4d). In addition, increased latency to groom in the ST (*t*_26_ = 6.145; *p* < 0.0001) (Fig. 4k) and to feed in the NSF (*t*_26_ = 3.082; *p* < 0.005) (Fig. 4l) were also observed. In the NSF, no differences in body weight before and after the testing session, or in home cage pellet consumption, were registered (Supplementary Fig. S4e,f). In the FST, increases in floating times were found (t_26_ = 4.411; *p* < 0.0002) (Fig. 4m). In contrast, no changes in locomotor activity were noted among experimental groups (*t*_26_ = 0.480; *p* = 0.64) (Fig. 4n).

Altogether, our results uncover a crucial role of 5-HTR7 in the developmental effects of FLX on adult anxiety and depressive-like behavior. Moreover, our findings indicate that the transient presence of 5-HTR7 in the PFC during early postnatal life is sufficient to largely replicate emotional alterations induced by FLX at this time period.

### Developmental 5-HTR7 blockade prevents detrimental paradoxical effects of SSRIs on emotional behavior

**Fig. 5:**
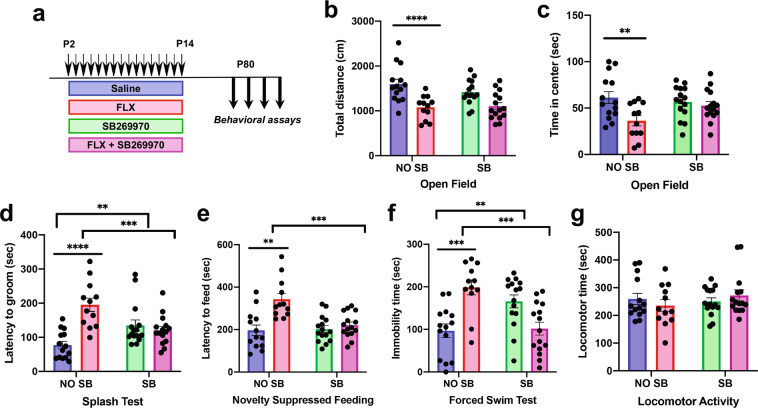
Developmental 5-HTR7 blockade prevents detrimental paradoxical effects of SSRIs on emotional behavior. **a** Four groups of C57BL/6 mice were administered subcutaneously with saline (*n* = 14), FLX (10 mg/kg/day; *n* = 12), FLX (10 mg/kg/day) + SB269970 (10 mg/kg/12 h) (*n* = 15), or SB269970 alone (10 mg/kg/12 h; *n* = 15) during the critical period (P2–P14) to evaluate adult emotional behaviors (from P80). Total distance traveled in the OF (**b**) (Main Effects SAL/FLX treatment: *F*_1,52_ = 23.289; *p* < 0.0001), and time spent in the center of the arena (**c**) (Main Effects SAL/FLX treatment: *F*_1,52_ = 7.708; *p* < 0.008). Latency to groom in the ST (**d**) (FLX/SAL x SB269970 treatment interaction: *F*_1,52_ = 20.406; *p* < 0.0001), latency to feed in the NSF (**e**) (FLX/SAL x SB269970 treatment interaction: *F*_1,51_ = 9.518; *p* < 0.003), and immobility time in the FST (**f**) (FLX/SAL x SB269970 treatment interaction: *F*_1,52_ = 26.952; *p* < 0.0001). Locomotor activity (**g**). **p < 0.01, ***p < 0.002, and ****p < 0.0001.

Next, we evaluated the potential benefit of antagonizing the 5-HTR7 during the postnatal FLX treatment to prevent the detrimental long-term effects on emotional behaviors. For this purpose, the specific 5-HTR7 antagonist SB269970 was co-administered with FLX during the critical period (from P2 to P14) in wild-type mice and emotional behaviors were investigated in adulthood (Fig. 5a). Two-way ANOVA results of the total distance traveled in the OF showed a non-significant interaction between FLX/SAL and SB269970 treatments (*F*_1,52_ = 1.463; *p* = 0.232). Consistent with previous studies, FLX treatment decreased the exploration in the OF in comparison to saline-treated mice (Main Effects: *F*_1,52_ = 23.289; *p* < 0.0001) (Fig. 5b), and this effect was not evident after SB269970 co-administration (Main Effects: *F*_1,52_ = 0.808; *p* = 0.37) (Fig. 5b). Time spent in the center of the arena showed an apparent interaction between treatments that did not reach significance (*F*_1,52_ = 3.985; *p* = 0.0512). Consistent to what happened with total distance traveled, FLX treatment decreased the time spent in the center (Main Effects: *F*_1,52_ = 7.708; *p* < 0.008), and this reduction was not longer visible when SB269970 was co-administered (Main Effects: *F*_1,52_ = 1.235; *p* = 0.27) (Fig. 5c). Total distance traveled in the center was not changed among all the experimental groups (Supplementary Fig. S4g).

In the ST, there was a significant interaction between both treatment factors (*F*_1,52_ = 20.406; *p* < 0.0001). Simple effects showed that the increased latency to groom produced by FLX when compared with control mice (*F*_1,24_ = 29.856; *p* < 0.0001) was completely prevented by concomitant treatment with SB269970 (*F*_1,25_ = 11.982; *p* < 0.002) (Fig. 5d). Interestingly, a less marked but significant increase in the latency to groom was observed in mice that had SB269970 alone (*F*_1,27_ = 8.401; *p* < 0.007) (Fig. 5d).

In the NSF we found a significant factor interaction in the latency to feed (*F*_1,51_ = 9.518; *p* < 0.003). Simple effect analyses showed that FLX increase in the latency to feed respect to saline controls (*F*_1,23_ = 15.845; *p* < 0.001), was prevented by SB269970 co-administration (*F*_1,25_ = 17.082; *p* < 0.0004) (Fig. 5e). On the other hand, no differences in body weight or in home cage pellet consumption were registered (Supplementary Fig. S4h, i).

Two-way ANOVA of the FST data showed a significant factor interaction (*F*_1,52_ = 26.952; *p* < 0.0001). Simple effect ANOVAs showed that increased floating time produced by FLX exposure respect to saline controls (*F*_1,24_ = 18.646; *p* < 0.0002) was prevented by SB269970 co-administration (*F*_1,25_ = 17.668; *p* < 0.0003) (Fig. 5f). In addition, similarly to what happened in the ST, an increased immobility time was observed in saline controls after SB269970 developmental treatment (*F*_1,27_ = 9.716; *p* < 0.004), suggesting a direct selective impact on depressive-like phenotypes (Fig. 5f). No treatment effects were detected in locomotor activity among all groups (Fig. 5g).

These results indicate a critical developmental role of 5-HTR7 in mediating the susceptibility to early postnatal FLX during the critical period. Furthermore, our findings demonstrate that concomitant systemic pharmacological antagonism of 5-HTR7 is sufficient to prevent the appearance of detrimental emotional effects caused by FLX in the early life.

## Discussion

This study identifies 5-HTR7 as a major player in prefrontal circuit development during the early postnatal period, with impact on adult emotional behaviors. We demonstrate that both morphological and behavioral consequences of a developmental exposure to SSRIs are dependent on the activation of the 5-HTR7 in the PFC. Importantly, the depressive-like and anxiety phenotypes caused by developmental FLX exposure can be prevented by the co-administration of a selective 5-HTR7 antagonist, opening interesting leads for modulation of antidepressant drug therapies during child-bearing ages.

In a previous study we identified the transient SERT expression in PFC neurons as an important target for developmental long-term detrimental effects of SSRIs [20]. Here, we found that 5-HTR7 is the 5-HT receptor with the highest expression in those cortical SERT+ neurons during early postnatal development. The transient expression of SERT and 5-HTR7 in the same neurons suggested the existence of a tight temporal control of 5-HT signaling through this receptor during this period. That is, the high affinity uptake of 5-HT mediated by SERT is expected to decrease 5-HT availability for 5-HTR7 at the neuronal cell membrane. Previous localization studies indicated a fairly broad distribution of 5-HTR7 in the adult brain including the cerebral cortex [44], but developmental studies are limited to only few brain regions in the rat [21, 45]. Our study showed that 5-HTR7 in the PFC has a transient expression in pyramidal neurons of layers 5–6 during the first 3 weeks of life, including SERT+ and SERT- neurons, while the expression in upper cortical layers is stable until adulthood. Consistent with our findings, Béïque et al. (2004) showed a dramatic shift in the effects of 5-HT on membrane potential over mPFC postnatal development. Specifically, during the first three postnatal weeks 5-HT induces a rapid depolarization of layer 5 pyramidal cells that can be largely blocked by 5-HTR7 antagonists but not by 5-HTR2A antagonists. Later in development, the characteristic 5-HTR1A-mediated adult hyperpolarization of the same pyramidal neurons produced by 5-HT can be only achieved after the third postnatal week [46, 47]. This shift in the physiological responses could be interpreted as a consequence of 5-HTR7 and 5-HTR1A dimerization, favoring the inhibitory component of the dimer [48, 49]. However, our data show that the 5-HTR7 expression in deep layer cortical neurons is down-regulated by P21, consistent with electrophysiology studies [46, 47].

Transient expression of 5-HTR7 in the PFC coincides with a period of high developmental plasticity. Pyramidal neurons of layers 5–6 in the mPFC send subcortical projections to a number of targets including the basal ganglia, amygdala, hypothalamus, and brainstem [40, 50]. Most of these projections are still undergoing active growth and synaptogenesis in subcortical targets during the first weeks of postnatal life. In particular, brainstem-projecting PFC neurons increase their inputs to the DRN establishing supernumerary excitatory synapses onto 5-HT and GABA neurons up to P21 [20]. The present experiments indicate that 5-HTR7 is an important player to modulate this synaptogenesis, since over-expression in the PFC increased the number of glutamatergic synapses in the DRN, while 5-HTR7^−/−^ mice had a reduced synaptic density. However, the chronic developmental application of the 5-HTR7 antagonist SB269970 alone did not modify the number of PFC glutamate synaptic afferents to the DRN. A possible interpretation of this difference is that in our pharmacological experiments the 5-HTR7 is only partially blocked  to the actions of 5-HT, especially when considering the narrow window of action of the SB269970 [31]. However, this partial action appeared sufficient to counterbalance the overgrowth of cortical glutamatergic synapses in the DRN produced by developmental exposure to FLX. It should be noted that estimates of glutamatergic synapses measured with array tomography could be affected by decreases in VGLUT1/synapsin expression levels. However, previous studies indicate a highly consistent electrophysiological functional correlate of the morphological synaptic measurements including those produced by developmental exposure to FLX [20, 37, 51].

5-HT is well known to have multiple morphogenetic roles, which differ according to 5-HT receptors and cell types [3, 52, 53]. Several in vitro studies concur to show that 5-HTR7 activation has a trophic effect, as 5-HTR7 agonists increased axon growth and synaptogenesis in hippocampal and striatal primary cultures [21, 23, 53]. The present study provides the first demonstration of an in vivo physiological effect of 5-HTR7 on a well-defined neural circuit. Compared with previous studies that focused on dendritic spine formation, we reveal here an additional morphogenetic effect on axon terminals, since manipulating 5-HTR7 expression in the PFC was sufficient to cause exuberance of glutamate terminals in the DRN. Moreover, the localization of 5-HTR7-GFP fusion protein at PFC axon terminals, strongly argues for a direct effect of 5-HTR7 stimulation at presynaptic sites, in addition to its previously identified postsynaptic effects on dendritic spine formation [21]. It will be interesting in the future to identify the downstream signaling mechanisms of this presynaptic effect, as 5-HTR7 is known to activate both Gs-cAMP-PKA and Gα12-RhoGAP-CDC42 pathways [21, 23, 53]. Our RNA profiling indicates that players of both pathways are present in the PFC-SERT+ neurons at P7 [20]. On a translational note, the demonstration that 5-HTR7 controls synaptogenesis and neural circuit development in the PFC, offers some interesting mechanistic insights to the proposal of *5-HTR7* gene variants as risk factors in neurodevelopmental psychiatric disorders such as schizophrenia [54] and autism spectrum disorders [55].

Exposure to a variety of SSRIs including FLX during the postnatal period has been repeatedly found to cause anxiety and depressive-like phenotypes later in life [20, 56–58]. Similarly, genetic models interfering with SERT function such as SERT-KO rodents display paradoxical depressive-like behaviors that can be explained by an increased 5-HT signaling during the first postnatal weeks [8, 59]. Pharmacological approaches indicated a partial role of the excess of activation of the 5-HTR1A [8] and 5-HTR2A receptors [58] in these effects. The present study provides complementary evidence pointing to a primordial role of the 5-HTR7 in the long-term emotional detrimental effects of developmental exposure to SSRIs. Depressive-like symptoms and anxiogenic responses were largely prevented by concomitant pharmacological treatment with a selective 5-HTR7 antagonist, and were substantially reduced in 5-HTR7^−/−^ mice receiving postnatal FLX. Interestingly, developmental blockade of 5-HTR7 by SB269970 *per se* produced adult depressive-like behavior but not anxiety phenotypes, in agreement with previous work showing a developmental effect of other 5-HT receptors like the 5-HTR2A/C in anxiety phenotypes [58]. It will be interesting to determine whether other behavioral symptoms that are due to developmental increases of 5-HT, such as sleep disturbances (increase in REM sleep) [8, 57] are also dependent on developmental effects of 5-HTR7, as this receptor is strongly expressed in the suprachiasmatic nucleus [44] and involved in the control of circadian rhythms and sleep [28, 60].

Present experiments showed that over-expression of the 5-HTR7 in PFC neurons was sufficient to induce anxiety and depressive-like symptoms in adulthood. Thus, developmental changes in PFC circuits are likely involved in their occurrence. However, it is difficult at present to pinpoint the relationship between specific circuit changes and emotional phenotypes. The PFC-to-DRN circuit that we focused on is an interesting candidate as it is recruited in stress control mechanisms and its general action appears to be that of dampening of 5-HT neuronal activity [17, 61–63]. Accordingly, stimulation of this pathway has been shown to have antidepressant effects [19], including in the postnatal FLX model [20]. Moreover, it is thought to underlie the beneficial effects of deep brain stimulation in treatment-resistant depressive patients [64]. Based on these studies, the synaptic exuberance of PFC terminals in the DRN can be interpreted as counteracting maladaptive circuit changes in other circuits. In this interpretation it should be noted that the PFC acts as a hub to control emotional behaviors by acting on a large number of brain targets with different outcomes according to targets. For instance top-down influence of the PFC on the midbrain dopaminergic neurons was proposed to cause anhedonia [65].

5-HTR7 antagonists have been proposed as a therapeutic approach in mood disorders [66] and could be an interesting alternative to SSRIs or combined to sub-threshold doses of SSRIs for the treatment of depression during pregnancy or postpartum. Treatment of severe depression in pregnant women remains a conundrum, because of the known risk to depressive mothers but also given the possible consequences of antidepressant treatments on child neurodevelopment [5, 67]. Current selective 5-HTR7 antagonists have a low bioavailability (because of a short half life and the requirement of parenteral administration) [31]. Interestingly, atypical neuroleptics such as Amisulpride [68] or Lurasidone [69], that have a high affinity for the 5-HTR7 acting as inverse agonists, were noted to have antidepressant effects. However, it remains to be determined whether these compounds would also be efficient mitigating the developmental detrimental consequences of SSRIs, as found here for SB269970.

## Funding and disclosure

The study was funded by the LabEx BioPsy and the Agence Nationale de la Recherche to PG (ANR-11-0004-02, ERA-NET Respond/ANR-15-0179, ANR-16-0162). JO benefited from a doctoral studentship from École des Neurosciences Paris Île de France. MS-R was supported by a Labex BioPsy fellowship and a 2015 NARSAD Young Investigator Grant from the Brain & Behavior Research Foundation (#23742). The authors declare no competing interests.

## Supplementary information

Supplementary Figure legends and Table

Supplementary Figure S1

Supplementary Figure S2

Supplementary Figure S3

Supplementary Figure S4
