# Supplementary material for: Implication of 5-HT7 receptor in prefrontal circuit assembly and detrimental emotional effects of SSRIs during development
Source: Neuropsychopharmacology. 2020 Jul 20;45(13):2267–77. doi: 10.1038/s41386-020-0775-z (PMC7784885; doi:10.1038/s41386-020-0775-z)
Supplement: Supplementary file 1 — Supplementary Figure legends and Table [file 41386_2020_775_MOESM1_ESM.docx]

**Supplementary material**

***Supplementary Table S1: List of primers used for genotyping and qPCR***

| *Gene* | *mRNA Variant* | *Product length* | *Forward Primer* | *Reverse Primer* |
| --- | --- | --- | --- | --- |
| *SCL6a4* | NM_010484.2 | 126 bp | TGG CAA CTG CAC CAA CTA CT | AGG TCC TGG AGT CCC TTT |
| *5-HTR7* | NM_008315.2 | 138 bp | TCG ACA GGT ACC TTG GGA TCA | TCT GAG CCC ATC CAA GAG A |
| *5-HTR7 exon 2* | NM_008315.2 | 400 bp | GAC AAA GTG TGC TTG ATC AGC CAG | ATG CAG CTA CAG GAG GTG CCA CAG |
| *GADPH* | NM_008084.3 | 150 bp | CTT CTT GTG CAG TGC CAG C | GAG GTC AAT GAA GGG GTC GT |

**Supplementary Figure S1:**

*In situ* hybridization of 5-HTR7 in the medial prefrontal cortex (mPFC) at P0 (**a**), P7 (**b**), P14 (**c**), P21 (**d**) and adults (**e,f**). A strong bilaminar expression of the receptor in layers 2 (arrowheads) and 5-6 (arrows) during the first two postnatal weeks is evidenced (**a-c**). 5-HTR7 expression within mPFC deep cortical layers start decreasing by P21 (**d**) to undetectable levels in adults (**e,f**). The 5-HTR7 expression strongly persist in adulthood in superficial layers of more lateral and caudal parts of the PFC, such as the anterior cingulate cortex (ACg, arrowhead) (**f**).

**Supplementary Figure S2:**

Transient over-expression of 5-HTR7 mRNA as revealed by qPCR analyses at P7 and P90 (adult) in the mPFC of C57BL/6 mice bilaterally injected with AAV-hSyn-5-HTR7-EGFP (OE) or AAV-hSyn-EGFP (Sham) at P1. *p<10^-5^ for P7-OE vs. all the other groups after ANOVA (F_3,8_ = 1815.773, p<10^-10^) followed by Tukey’s comparisons.

**Supplementary Figure S3:**

Behavioral analyses of the 5-HTR7 ^+/+^, 5-HTR7 ^+/-^ and 5-HTR7 ^-/-^ mice. Littermates of both genders were analyzed in the Open Field test (OF), Novelty Suppressed Feeding test (NSF), Splash test (ST), Forced Swim test (FST) and for locomotor activity. Data were evaluated by ANOVA. Total distance (**a**) (F_2,25_ = 0.591; p = 0.56), total distance in the center (**b**) (F_2,25_ = 1.171; p = 0.32), and time in the center (**c**) (F_2,25_ = 0.074; p = 0.93) were registered in the OF. Home cage food consumption (**d**) (F_2,25_ = 0.443; p = 0.65), latency to feed (**e**) (F_2,25_ = 0.302; p = 0.74), and weight loss (**f**) (F_2,25_ = 0.927; p = 0.41) were analyzed in the NSF. Latency to groom in the ST (**g**) (F_2,25_ = 0.916; p = 0.41), immobility time in the FST (**h**) (F_2,25_ = 2.917; p = 0.07), and locomotor activity (**i**) (F_2,25_ = 0.252; p = 0.78) are also shown.

**Supplementary Figure S4:**

Behavioral analyses of 5-HTR7 ^+/-^ and 5-HTR7 ^-/-^ mice treated orally with FLX (10 mg/kg/day, n=16 and n=22 for 5-HTR7 ^+/-^ and 5-HTR7 ^-/-^ mice, respectively) or vehicle (3% sucrose solution; n=14 and n=24 for 5-HTR7 ^+/-^ and 5-HTR7 ^-/-^ mice, respectively) from P2 to P14. All behavioral assessments started at P80. **a)** Total distance traveled in the center of the OF arena (Main Effects treatment: F_1,72_ = 0.207; p = 0.65). **b-c)** The percentage of weight loss (**b**) and food consumption in the home cage (**c**) at the NSF (Main Effects treatment: F_1,72_ = 1.114; p = 0.30 and F_1,72_ = 0.883; p = 0.35, respectively). **d-f)** Adult behavioral measurements of C57BL/6 mice bilaterally injected with AAV-hSyn-5-HTR7-EGFP (OE; n=14) or AAV-hSyn-EGFP (Sham; n=14) in the PFC at P1. Total distance traveled in the center of the arena (**d**) (t_26_ = 1.751; p = 0.09). **e-f)** Percentage of weight loss (**e**) (t_26_ = 1.492; p = 0.15) and food consumption in the home cage (**f**) (t_26_ = 1.339; p = 0.19) in the NSF. **g-i)** Adult behavior of C57BL/6 mice administered subcutaneously with saline (n=14), FLX (10 mg/kg/day; n=12), FLX (10mg/kg/day) + SB269970 (10mg/kg/12hs) (n=15), or SB269970 alone (10mg/kg/12hs; n=15) during the critical period (P2 to P14). Total distance traveled in the center of the OF arena (**g**), and percentage of weight loss (**h**) and home cage pellet consumption (**i**) in the NSF are shown.
