# Supplementary figures and images for: Implication of 5-HT7 receptor in prefrontal circuit assembly and detrimental emotional effects of SSRIs during development
Source: Neuropsychopharmacology. 2020 Jul 20;45(13):2267–77. doi: 10.1038/s41386-020-0775-z (PMC7784885; doi:10.1038/s41386-020-0775-z)

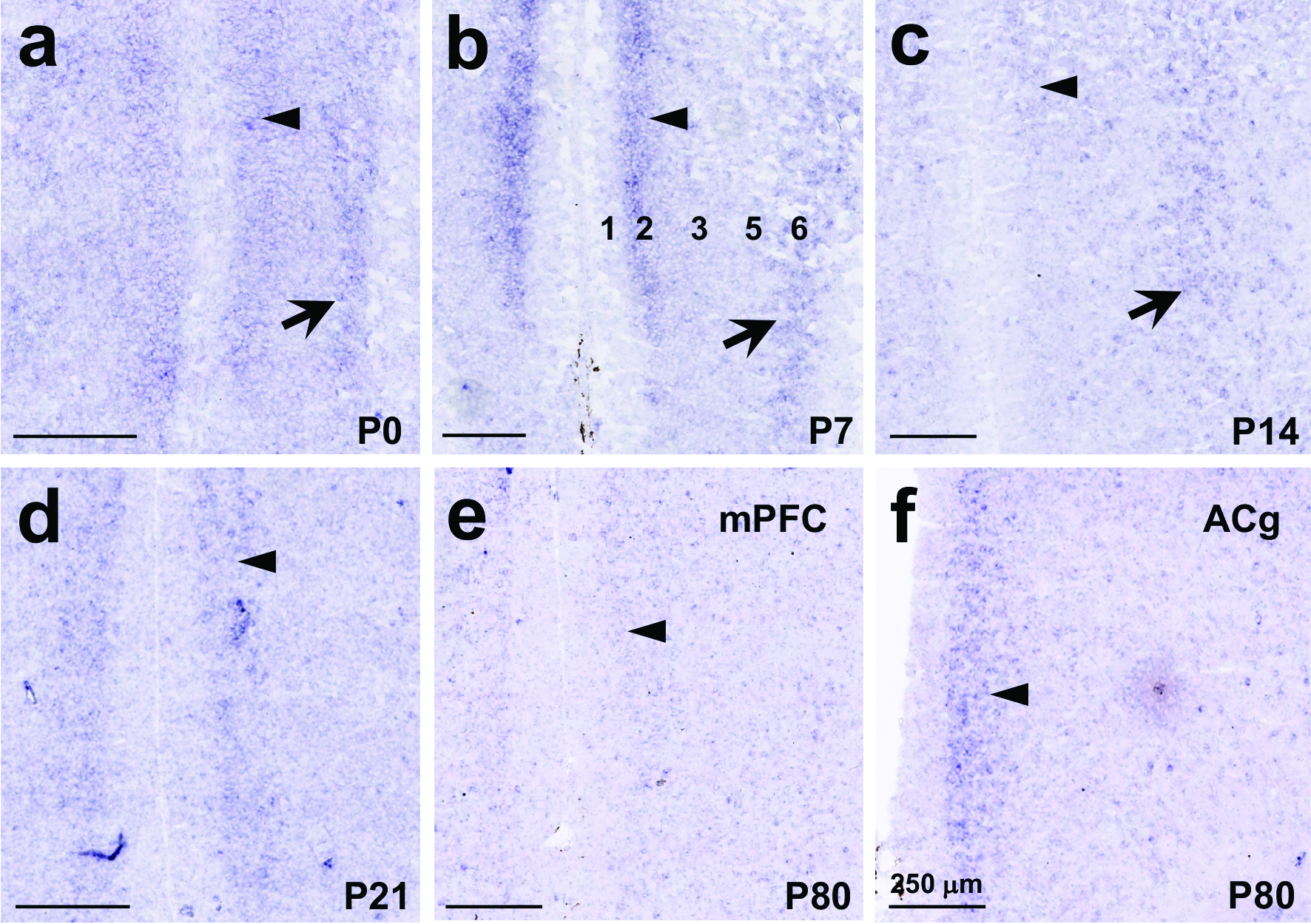

Supplement: Supplementary file 2 — Supplementary Figure S1 [file 41386_2020_775_MOESM2_ESM.jpg]

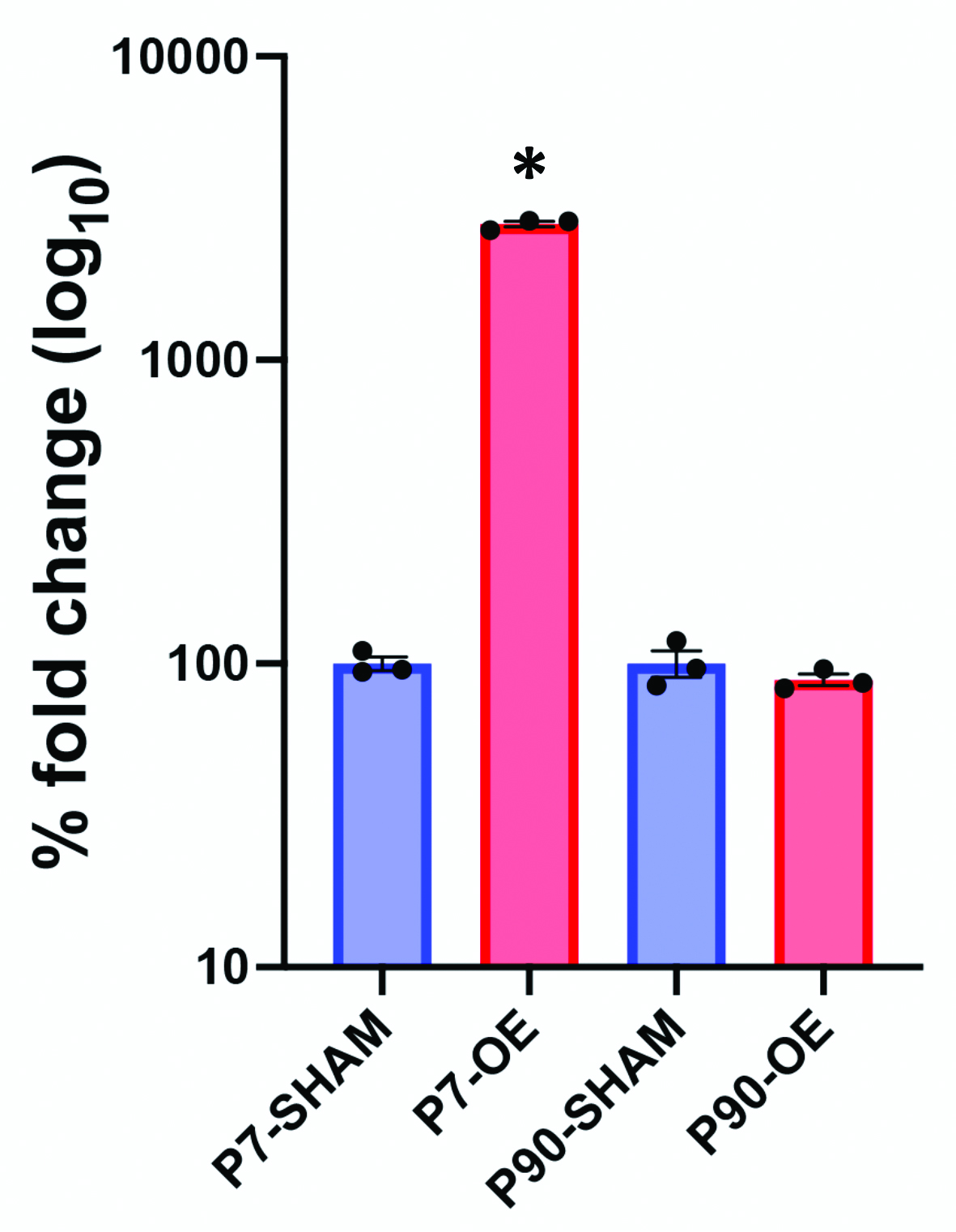

Supplement: Supplementary file 3 — Supplementary Figure S2 [file 41386_2020_775_MOESM3_ESM.jpg]

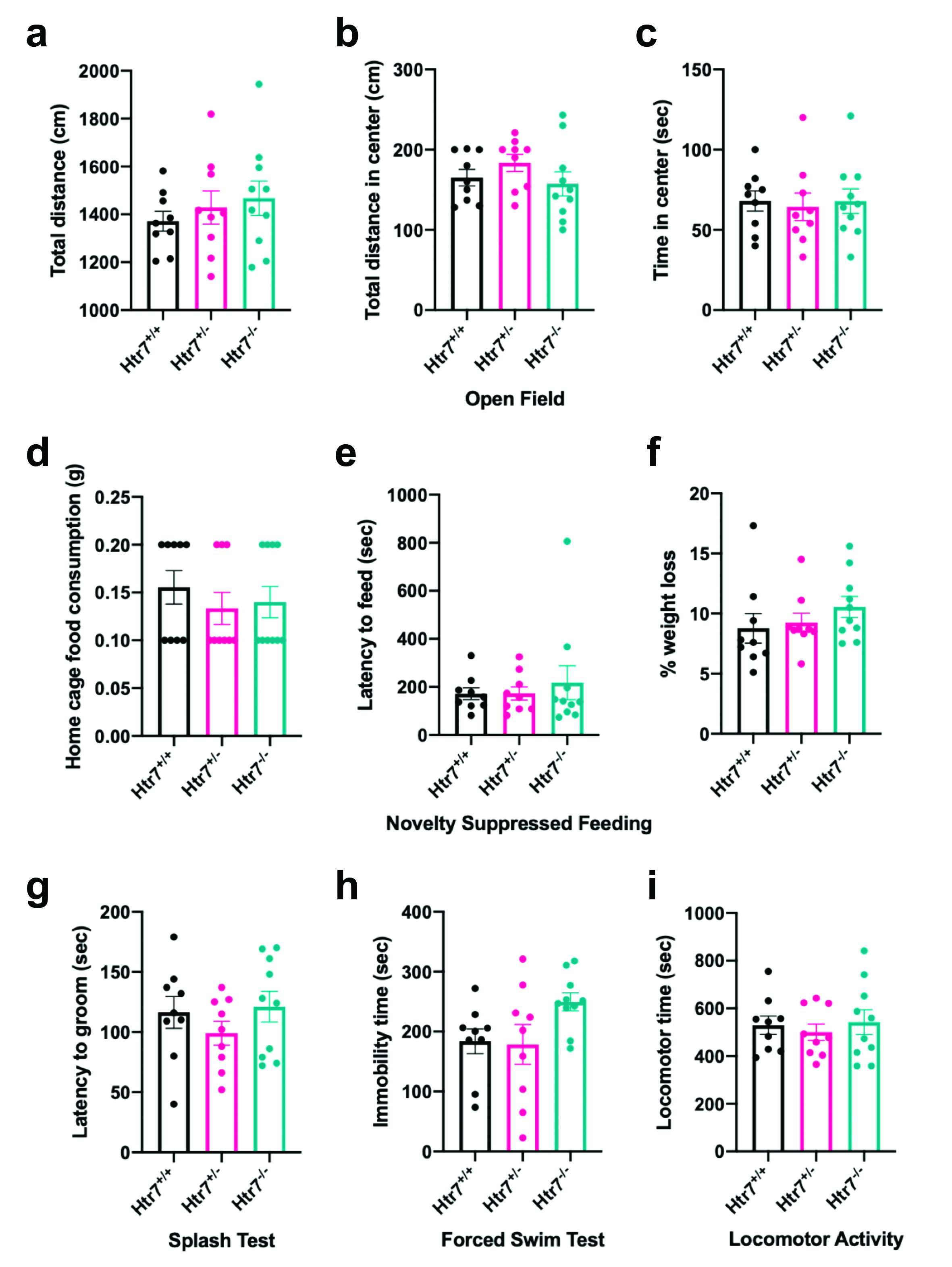

Supplement: Supplementary file 4 — Supplementary Figure S3 [file 41386_2020_775_MOESM4_ESM.jpg]

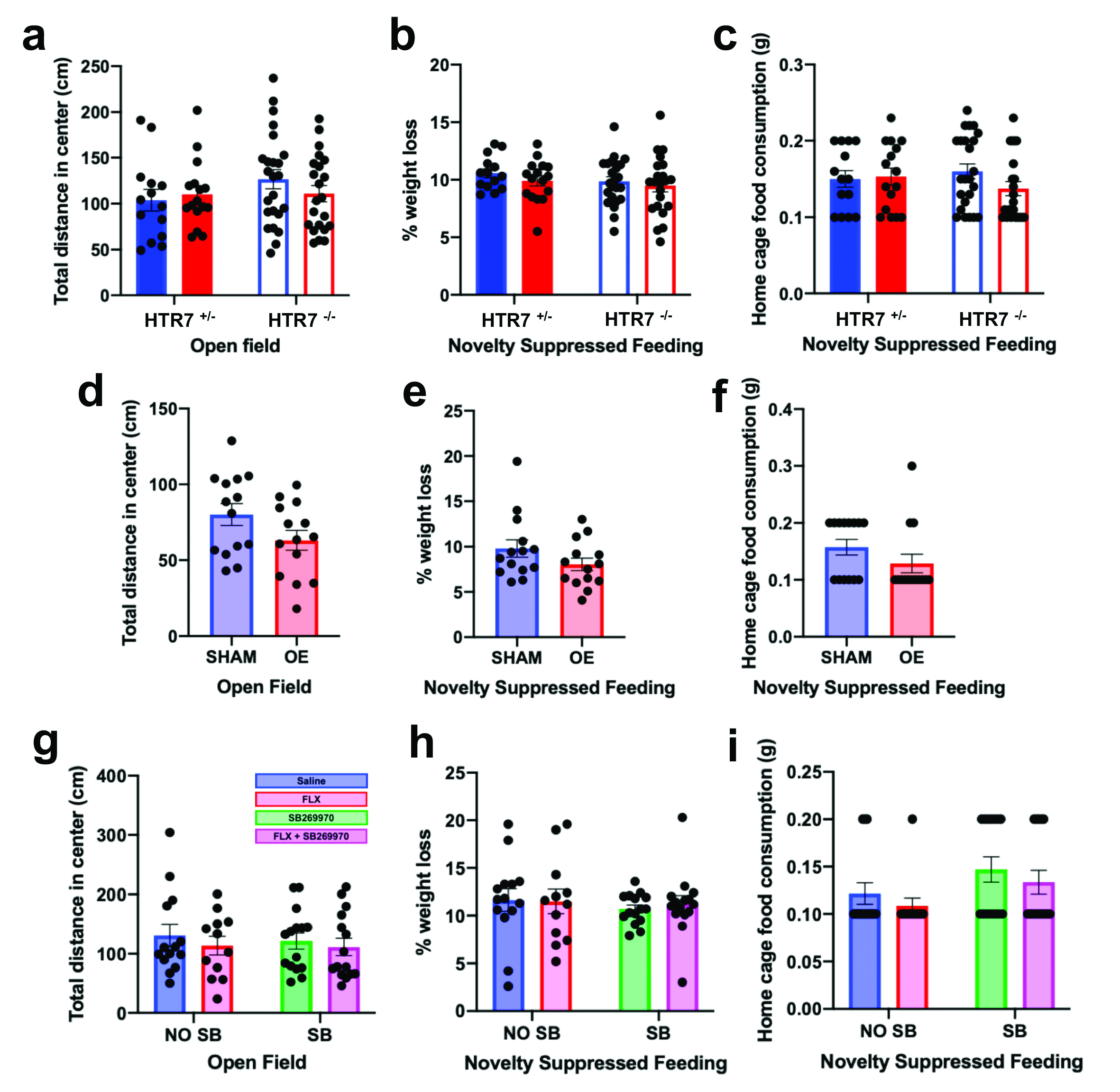

Supplement: Supplementary file 5 — Supplementary Figure S4 [file 41386_2020_775_MOESM5_ESM.jpg]
